# Characterization of vB_SauM-fRuSau02, a Twort-Like Bacteriophage Isolated from a Therapeutic Phage Cocktail

**DOI:** 10.3390/v9090258

**Published:** 2017-09-14

**Authors:** Katarzyna Leskinen, Henni Tuomala, Anu Wicklund, Jenni Horsma-Heikkinen, Pentti Kuusela, Mikael Skurnik, Saija Kiljunen

**Affiliations:** 1 Department of Bacteriology and Immunology, Medicum, Research Programs Unit, Immunobiology Research Program, University of Helsinki, Helsinki 00290, Finland; katarzyna.leskinen@helsinki.fi (K.L.); henni.tuomala@helsinki.fi (H.T.); anumaria.wicklund@gmail.com (A.W.); jenni.horsma@helsinki.fi (J.H.-H.); mikael.skurnik@helsinki.fi (M.S.); 2 Division of Clinical Microbiology, HUSLAB, University of Helsinki and Helsinki University Hospital, Helsinki 00290, Finland; pentti.kuusela@hus.fi

**Keywords:** *Staphylococcus aureus*, bacteriophage, phage therapy, vB_SauM-fRuSau02, *Twortlikevirus*

## Abstract

*Staphylococcus aureus* is a commensal and pathogenic bacterium that causes infections in humans and animals. It is a major cause of nosocomial infections worldwide. Due to increasing prevalence of multidrug resistance, alternative methods to eradicate the pathogen are necessary. In this respect, polyvalent staphylococcal myoviruses have been demonstrated to be excellent candidates for phage therapy. Here we present the characterization of the bacteriophage vB_SauM-fRuSau02 (fRuSau02) that was isolated from a commercial *Staphylococcus* bacteriophage cocktail produced by Microgen (Moscow, Russia). The genomic analysis revealed that fRuSau02 is very closely related to the phage MSA6, and possesses a large genome (148,464 bp), with typical modular organization and a low G+C (30.22%) content. It can therefore be classified as a new virus among the genus *Twortlikevirus*. The genome contains 236 predicted genes, 4 of which were interrupted by insertion sequences. Altogether, 78 different structural and virion-associated proteins were identified from purified phage particles by liquid chromatography-tandem mass spectrometry (LC-MS/MS). The host range of fRuSau02 was tested with 135 strains, including 51 and 54 *Staphylococcus aureus* isolates from humans and pigs, respectively, and 30 coagulase-negative *Staphylococcus* strains of human origin. All clinical *S. aureus* strains were at least moderately sensitive to the phage, while only 39% of the pig strains were infected. Also, some strains of *Staphylococcus intermedius*, *Staphylococcus lugdunensis*, *Staphylococcus epidermidis*, *Staphylococcus haemolyticus*, *Staphylococcus saprophyticus* and *Staphylococcus pseudointer* were sensitive. We conclude that fRuSau02, a phage therapy agent in Russia, can serve as an alternative to antibiotic therapy against *S. aureus*.

## 1. Introduction

*Staphylococcus aureus* is a commensal and pathogenic bacterium that causes opportunistic infections in humans and animals. Approximately 20% of humans have persistent and 30% sporadic nasal *S. aureus* colonization [1]. As a pathogen, *S. aureus* causes a broad spectrum of infections in humans ranging from simple abscesses to fatal sepsis, including pneumonia, endocarditis, meningitis, mastitis, food poisoning, and toxic shock syndrome [2]. Currently the antibiotic resistance of this species poses a threat to public health. Even though the incidence of severe infections caused by methicillin-resistant *S. aureus* (MRSA) is decreasing [3], MRSA still is an important cause of nosocomial infections worldwide [4,5]. The emergence of multidrug resistance results in difficulties in eradication of the pathogen with the use of conventional therapies and thus requires development of alternatives to antibiotic-based therapies.

One promising alternative to treat infections caused by antibiotic resistant bacteria is phage therapy, where the natural predators of bacteria (bacteriophages, phages) are used to kill the pathogens [6,7,8]. The history of phage therapy has been extensively reviewed elsewhere [9,10] and is not discussed here. To be considered safe, phage therapy has to meet a number of criteria: phages used for therapeutic purposes need to be strictly lytic and they should not carry known genes coding for toxins or other harmful substances [11]. Furthermore, the host bacteria used for phage production should have as few prophages as possible and the therapeutic phage preparation should not contain high concentration of bacterial toxins.

All known *S. aureus* phages belong to order *Caudovirales*, i.e., they are tailed phages with an icosahedral capsid that surrounds the double-stranded DNA genome [12,13]. Staphylococcal phages can be classified into three categories: (1) podoviruses with <20 kb genomes; (2) siphoviruses with ~40 kb genomes; and (3) myoviruses with >125 kb genomes [12]. Of these phage groups, staphylococcal siphoviruses are generally temperate and often carry genes promoting bacterial virulence [13], which makes them inappropriate for therapeutic applications. Staphylococcal podoviruses, on the other hand, are strictly lytic but extremely rare and difficult to find [14]. From therapeutic point of view, myoviruses are considered the most interesting staphylococcal phages [14,15].

Many of the staphylococcal myoviruses are classified into the genus *Twortlikevirus* of the *Spounavirinae* subfamily and are related at genetic and proteomic level [16]. The *Twortlikevirus* genus consists of phages with genomes of 127–141 kb, low G+C content (30–31%), and 183 to 217 open reading frames (ORFs) [17]. Currently, this genus contains over 25 members, including phage Twort, G1 [18], K [19], MSA6 [20], GH15 [21], Romulus, and Remus [17]. A typical feature for Twort-like viruses is the presence of long terminal repeats (LTRs), several thousand base pair-long direct repeats at the ends of the genome. The nucleotide sequence and length of LTR regions differ among the representatives of the genus and may influence the host range [15]. Twort-like viruses are also known for their broad host range. This phenomenon is mainly accounted to the presence of multiple receptor binding proteins in the viral capsid that allow them to utilize at least two adsorption apparatuses and recognize different structures [22]. This feature, together with their strictly lytic life cycle, makes Twort-like viruses particularly suitable for clinical applications [15].

In this paper, we report the isolation and analysis of a Twort-like *S. aureus* phage, vB_SauM-fRuSau02 (fRuSau02). The phage was isolated from a therapeutic bacteriophage product from Microgen Company (Moscow, Russia). The product was purchased in a pharmacy in Saint Petersburg, Russia, and was meant to treat infections typically caused by *S. aureus*. However, no information about the phage composition or the efficacy of the phage cocktail was available. Phage fRuSau02 was the only phage we were able to isolate from this product. Here, we show the analysis of fRuSau02 at a genetic and proteome level, the latter of which allowed us to identify the majority of the phage structural proteins. Additionally, we provide an insight into the reasons why this phage might be well-suited for clinical applications by testing its growth efficiency and host range with a broad range of human and porcine isolates. We also present an evaluation of the fRuSau02 production in different host strains, with intention to select an optimal producer strain for clinical applications.

## 2. Materials and Methods

### 2.1. Bacterial Strains, Phages and Media

The bacterial strains used in this work are described in Table S1. The collection of human isolates used in this study was provided by The Hospital District of Helsinki and Uusimaa Laboratories (HUSLAB), Finland. All staphylococcal and phage incubations were done at 37 °C using Luria Broth (LB) [23] medium. Soft agar medium included additionally 0.35 or 0.4% (*w*/*v*) agar (Becton Dickinson, Franklin Lakes, NJ, USA), and LB agar plates were solidified with 1.5% (*w*/*v*) of agar. fRuSau02 was isolated using a clinical *S. aureus* strain 13KP (Table S1) as a host, and the same strain was then used as a standard host strain for the phage. The phage lysates were produced from semiconfluent soft-agar plates as described elsewhere [23].

### 2.2. Phage Purification

The fRuSau02 lysate (5 × 10^10^ plaque-forming units (PFU)/mL) was ultrafiltrated with Amicon Ultra-4 (100 kDa) Centrifugal Filter Units (Merck Millipore, Billerica, MA, USA) to one quarter of the initial volume. Three volumes of chromatography buffer A (20 mM Tris-Cl, pH 7.5) were added and the ultrafiltration was repeated. The volume was adjusted with buffer A. The ultrafiltrated phage sample was then purified with ion exchange chromatography (IEX) using Äkta Purifier (GE Healthcare, Chicago, IL, USA) and a CIM QA-1 tube monolithic column with a 6-µm pore size (BIA Separations, Ajdovščina, Slovenia). The sample was injected to the column in buffer A, washed with buffer A containing 350 mM NaCl and eluted with buffer A with 450 mM NaCl. The phage-containing fractions of two purification batches were pooled, and an Amicon Ultra was used to concentrate the product and to change the buffer to TM (50 mM Tris, pH 7.5–10 mM Mg_2_SO_4_). Purified phage samples were stored at 4 °C.

### 2.3. Electron Microscopy

IEX-purified phage lysate was pelleted by centrifugation at 16,000× *g*, 4 °C, for 2 h and resuspended into 0.1 M ammonium acetate. Subsequently, the phage particles were allowed to sediment on 200 mesh pioloform-coated copper grids for 1 min and stained negatively using 3% uranyl acetate. Samples were examined with a JEOL JEM-1400 transmission electron microscope JEOL Ltd., Tokyo, Japan) under 80 kV at the Electron Microscopy Unit (Institute of Biotechnology, University of Helsinki, Helsinki, Finland). Pictures were taken using Gatan Orius SC 1000B bottom-mounted Charged Coupled Device (CCD)-camera (Gatan Inc., Pleasanton, CA, USA). Ten virions were measured and data were used to calculate mean values and standard deviations.

### 2.4. Infection Growth Curves

Overnight bacterial cultures of *S. aureus* 13KP were diluted to a ratio of 1:100 in fresh LB medium, and 180-µL aliquots were distributed into honeycomb plate wells (Growth Curves Ab Ltd., Helsinki, Finland), where they were mixed with 20-µL aliquots of different fRuSau02 phage stock dilutions. The phage stock and bacterial culture were mixed to achieve multiplicity of infection (MOI) values ranging between 5 × 10^−7^ and 500. A negative control was obtained by mixing 20 µL of phage stock with 180 µL of LB, whereas the positive control consisted of 180 µL of bacterial culture and 20 µL of fresh LB medium. The growth experiment was carried out at 37 °C using a Bioscreen C incubator (Growth Curves Ab Ltd.) with continuous shaking. The optical density at 600 nm (OD_600_) of the cultures was measured every 1 h. The averages were calculated from values obtained for the bacteria grown in five parallel wells.

### 2.5. DNA Isolation and Phage Genome Sequencing

fRuSau02 DNA was isolated from crude phage lysate with Invisorb Spin Virus DNA Mini Kit (Stratec Biomedical, Birkenfeld, Germany). Sequencing was performed at the Institute for Molecular Medicine Finland (FIMM) Technology Centre Sequencing Unit [24]. For next-generation sequencing, the DNA library was constructed with Nextera sample prep kit (Illumina, San Diego, CA, USA). Paired-end sequencing was done using Illumina MiSeq PE300 sequencer (Illumina, San Diego, CA, USA) with the read length of 300 nucleotides. TheA5 (Andrew And Aaron’s Awesome Assembly)-miseq integrated pipeline for de novo assembly of microbial genomes was used to obtain the genome sequence [25]. fRuSau02 sequence was submitted to GenBank with accession number MF398190.

### 2.6. Determination of Physical Ends of the Phage Genome

To determine the physical ends of the phage genome, the approximate positions of the terminal repeats were estimated based on the sequence read numbers using the Integrative Genomics Viewer (IGV) [26,27]. The genome was manually edited according to the estimated physical ends and subjected to virtual digestions with several restriction endonucleases. Two enzymes (NheI and PstI), yielding identifiable end fragments, were used to digest the fRuSau02 DNA, and the resulting fragment distributions were compared to the virtual digestions. The NheI fragments corresponding to the physical ends of the phage genome were isolated from a preparative agarose gel and ligated to pUC19 digested with XbaI and SmaI. The ligation mixtures were used as a template for PCR reaction with pUC19—specific primers Puc19-F (CCTCTTCGCTATTACGCCAG) and pUC19-R (CAACGCAATTAATGTGAGTTAGCT). The PCR products corresponding to the sizes of the genome end fragments (2.7 kb and 4.5 kb for left and right ends, respectively) were isolated from a preparative agarose gel and sequenced with ABI3730XL DNA Analyzer (Applied Biosystems, Foster City, CA, USA) capillary sequencer with primers Puc19-F and Puc19-R at FIMM [24]. The sequence information was used to deduce the actual sequence of the genome ends and terminal repeats.

### 2.7. In Silico Analysis of Phage Genome

The phage genome was autoannotated using Rapid Annotation Using Subsystem Technology (RAST [28] and proofread manually. Promoters and terminators were predicted using PePPER [29] and ARNold [30,31], respectively, with subsequent manual verification. The promoter consensus sequence was analyzed using MEME [32]. A comparative genome figure was generated using CGView [33]. The genome-wide comparison of bacteriophages was conducted with EMBOSS Stretcher [34].

Phylogeny analysis was conducted with the VICTOR Virus Classification and Tree Building Online Resource [35] using the Genome-BLAST Distance Phylogeny (GBDP) method [36] under settings recommended for prokaryotic viruses [35]. The resulting intergenomic distances (including 100 replicates each) were used to infer a balanced minimum evolution tree with branch support via FASTME including Subtree Pruning and Regrafting (SPR) postprocessing [37] for the formula D0. The tree was rooted at the midpoint [38] and visualized with FigTree [39]. Taxon boundaries at the species, genus and family level were estimated with the OPTSIL program [40], the recommended clustering thresholds [35] and an F value (fraction of links required for cluster fusion) of 0.5 [41].

### 2.8. Proteome Analysis

IEX-purified phages were concentrated by centrifugation for 2 h at 4 °C and 16,000× *g*. Prior to digestion of proteins to peptides with trypsin, the proteins in the samples were reduced with tris(2-carboxyethyl)phosphine (TCEP) and alkylated with iodoacetamide. Tryptic peptide digests were purified by C18 reversed-phase chromatography columns [42] and the mass spectrometry (MS) analysis was performed on an Orbitrap Elite Electron-Transfer Dissociation (ETD) mass spectrometer (Thermo Scientific, Waltham, MA, USA), using Xcalibur version 2.7.1, coupled to an Thermo Scientific nLCII nanoflow High Pressure Liquid Chromatography (HPLC) system. Peak extraction and subsequent protein identification was achieved using Proteome Discoverer 1.4 software (Thermo Scientific). Calibrated peak files were searched against the fRuSau02 and *Staphylococcus aureus* subsp. *aureus* ST398 proteins (ASM188707v1, NCBI) by a SEQUEST search engine. Error tolerances on the precursor and fragment ions were ±15 ppm and ±0.6 Da, respectively. For peptide identification, a stringent cut-off (0.5% false discovery rate) was used. The LC-MS/MS was performed at the Proteomics Unit, Institute of Biotechnology, University of Helsinki.

### 2.9. Host Range Screening

The fRuSau02 host range was analyzed by spot assay for most of the bacterial stains studied. Some pig isolates failed to grow on soft agar, and their sensitivity was studied by a liquid culture method. For this, bacteria were cultured overnight in Brain Heart Infusion (BHI) medium (Becton, Dickinson and Company, Franklin Lakes, NJ, USA). Cultures were diluted 1:100 in BHI and aliquoted into 200 µL aliquots to 96-well plates. To these, 10 µL of phage fRuSau02 (6.8 × 10^6^ PFU) was added and the plate was incubated at 37 °C with moderate shaking. For non-infected controls, 10 µL of LB was added instead of the phage. Each strain was studied in triplicate wells. Bacterial growth was monitored by measuring OD_600_ with FLUOstar OPTIMA plate reader (BMG LABTECH GmbH, Ortenberg, Germany) at 60 min intervals for 4 h, and the inhibition of growth in phage-infected wells compared to non-infected control wells indicated a sensitive strain.

### 2.10. Efficiency of Plating and Adsorption Assay

Bacterial strains were checked by the efficiency of plating (EOP), as described earlier [43]. In brief, *S. aureus* strains 13KP, Newman, TB4, and tagO were pre-grown for 2–3 h at 37 °C. Subsequently 300 PFU of phage was mixed with 90 µL/OD_600_ of bacterial culture and 3 mL of soft agar (0.35% LB agar) and poured over an LB plate. Following 24 h incubation at 37 °C, PFUs were counted and the size and morphology of the plaques was evaluated. The experiment was performed in triplicates, and negative controls without the bacteriophages were prepared. To estimate the adsorption of phage particles on the surface of different *S. aureus* strains, a phage adsorption assay was conducted as described earlier [44]. Briefly, approximately 2.5 × 10^6^ PFU of fRuSau02 was mixed with 500 µL of bacterial overnight cultures (OD_600_ = 3.3). The suspension was incubated at room temperature for 5 min, centrifuged at 16,000× *g* for 3 min, and the phage titer remaining in the supernatant was determined. The phage titer in the control supernatant was set to 100%. LB was used as a non-adsorbing control. Each assay was performed in triplicates.

### 2.11. Staphylococcal Enterotoxin Measurement

Staphylococcal enterotoxins were measured from the phage lysates with the Transia Plate Staphylococcal Enterotoxins assay (BioControl Systems, Inc., Bellevue, WA, USA) using staphylococcal enterotoxin A (Sigma-Aldrich, St. Louis, MO, USA) as standard. Absorbance at 450 nm was recorded with Hidex Sense Microplate Reader (Hidex, Turku, Finland).

## 3. Results

### 3.1. Isolation and Morphology

**Figure 1:**
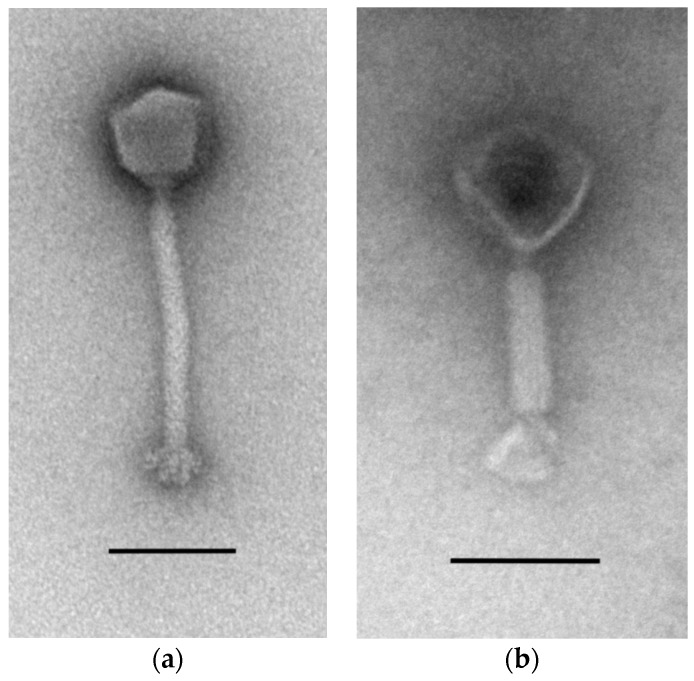
Electron micrographs of negatively stained vB_SauM-fRuSau02 (fRuSau02) particles. Phage particles with non-contracted (**a**) and contracted (**b**) tails are shown. Bars represent 100 nm.

Phage fRuSau02 was isolated from the *Staphylococcus* bacteriophage cocktail produced by the Microgen Company (Moscow, Russia; series: H52, 0813, PN001973/01). Electron microscopy of the negatively-stained fRuSau02 particles revealed that the phage had an icosahedral head with a contractile tail and a basal tuft attached to the tail (Figure 1). The dimensions of the head were 86 nm (vertical) and 83 nm (horizontal), and the tail length without the base plate was 192 nm. Standard deviations were 3.1, 2.8, and 5.3 nm respectively. Only one particle with contracted tail was found (Figure 1b), and the contracted part was 96 nm. Based on the morphological characteristics, phage fRuSau02 belongs to the order *Caudovirales* and the family *Myoviridae* [45,46].

### 3.2. The Efficiency of Infection

**Figure 2:**
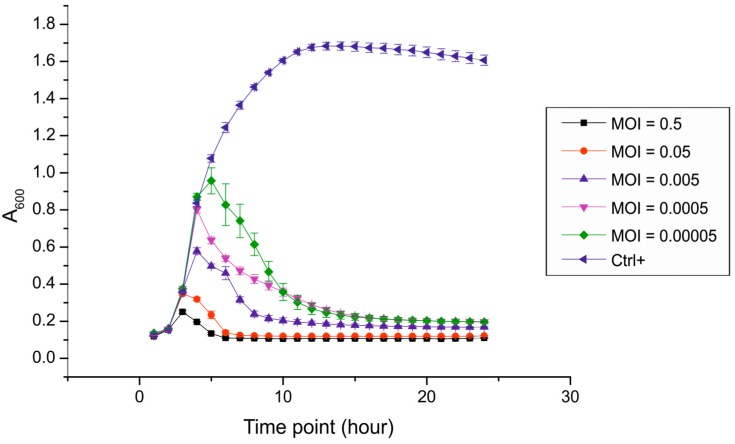
Growth curves of *Staphylococcus aureus* of 13KP infected with fRuSau02. Bacteria were cultured with different concentrations of phage virions in Luria Broth (LB) at 37 °C. Each curve represents the average results for five replicates, error bars represent standard deviation (SD). MOI: multiplicity of infection.

To examine the efficiency of fRuSau02 infection, *S. aureus* 13KP was infected in liquid culture at different MOI values and the bacterial growth was assessed by following the optical density of the culture. The study showed that fRuSau02 is able to efficiently lyse the culture at MOIs above 5 × 10*^−^*^5^ (Figure 2), whereas at MOIs values below this limit there was no lysis observed (data not shown). Additionally, there was no re-growth of the bacterial culture observed within the 24 h time period of the experiment. In coherence, the prolonged incubation of the infected bacteria in the soft agar did not result in emergence of resistance within the first 7 days of incubation indicating low rate of phage-resistance development among the bacteria. Efficient infection and complete lysis of bacterial culture with very low MOI values may be a common feature of twort-like phages, as MOI 1 × 10*^−^*^4^ was earlier shown to be optimal for the production of high-titer lysate of phage MSA6 [20].

### 3.3. General Genome Analysis

**Figure 3:**
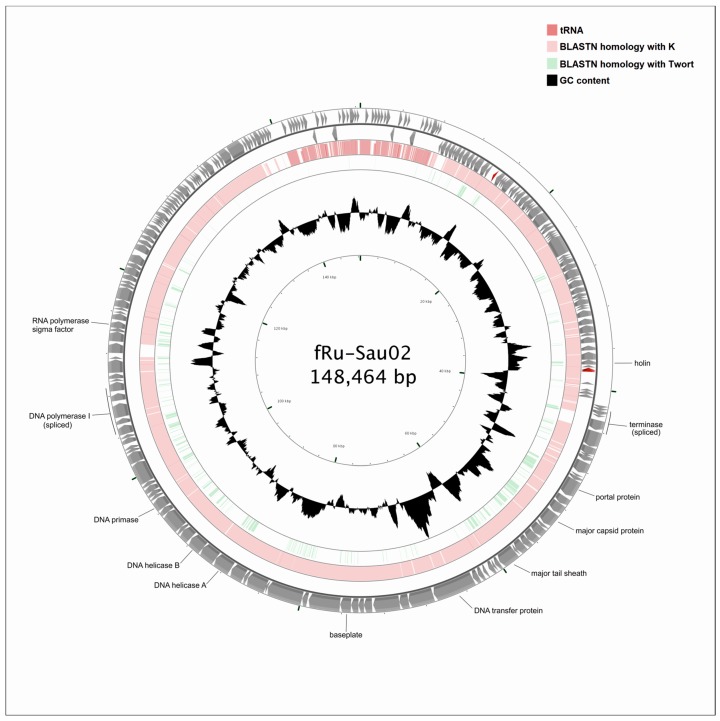
Genome comparison of three Twort-like phages. The outer ring represents the open reading frames (ORFs) of the circularized fRuSau02 phage. The two other rings display the identity between fRuSau02 and K (lavender) and between fRuSau02 and Twort (green). The inner ring shows the GC content of the fRuSau02 genome (black). Selected gene functions are indicated. The figure was generated with CGView [33].

**Figure 4:**
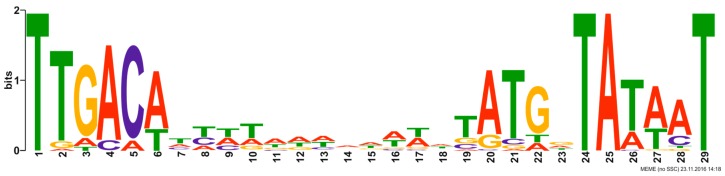
Consensus sequence of the phage fRuSau02 putative promoters. The promoter sequences are listed in supplementary Table S2.

The linear double-stranded DNA of fRuSau02 comprises 148,464 bp encoding 236 putative ORFs (Figure 3, Table 1). The two terminally redundant 8076 bp long ends encode 20 putative terminal repeat proteins. Sixty-five of the predicted genes are transcribed from the minus strand, including the genes likely to be involved in bacterial cell lysis (holin and numerous putative membrane proteins). Additionally, two single genes from the terminally redundant region, *treI* and *treM*, are also encoded on the minus strand. *In silico* analysis predicted the presence of 43 bacterial promoters and 32 terminators. The analysis failed to identify promoters within the 39,000–66,500 bp range in the fRuSau02 genome where the structural proteins are encoded. The consensus sequence of the promoters was identified (Figure 4 and Table S2). Interestingly, three predicted promoters located in front of spliced or intron encoded genes (*lysK.1*, *ksaI*, *I-MsaI*) presented a distinctive promoter sequence that did not follow the consensus sequence (Table S2). In addition to protein-coding sequences, three functional transfer RNA(tRNA) genes encoding tRNA^Met^, tRNA^Phe^, and tRNA^Asp^ were detected. Additionally, no known genes encoding integrases, lysogeny- or virulence-associated or toxic proteins were identified and therefore this bacteriophage can be considered as virulent and potentially safe for phage therapy. Similarly to phage K, the genome of fRuSau02 completely lacks GATC sites that could be recognized by host-encoded restriction endonucleases [19].

### 3.4. Comparative Genome Analysis

**Figure 5:**
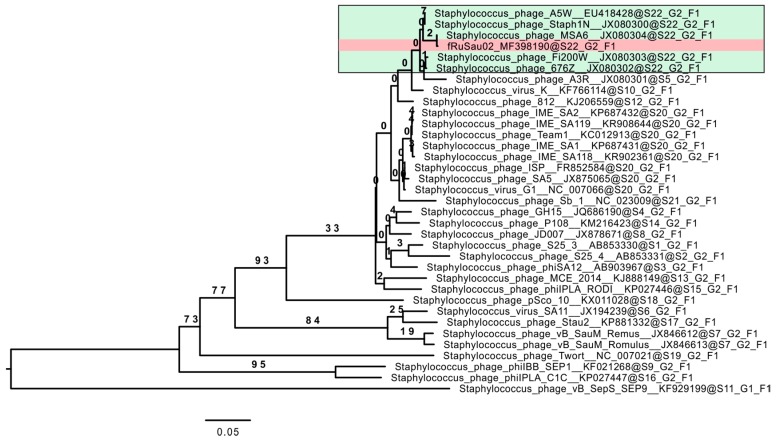
Genome-wide nucleotide phylogeny of 35 Twort-like viruses. The analysis was conducted with VICTOR Virus Classification and Tree Building Online Resource [35] with settings recommended for prokaryotic viruses. The tree was visualized with FigTree [39]. The analysis yielded 22 clusters at species (S1–S22) and two at genus (G1–G2) level, respectively. All the phages clustered to the same family (F1). Phage fRuSau02 is indicated with red box and the phages belonging to the same species with it in the green box.

Bioinformatic analysis of the fRuSau02 genome revealed that the phage has a genome size and organization typical for Twort-like viruses [15]. It is most closely related to phage MSA6 (JX080304), the two viruses showing 99.6% identity at the nucleotide level. The DNA sequence comparison with other Twort-like viruses revealed identity in the range of 39.0–96.0% (Table S3). The highest identity was observed with phages A5W (EU418428)—96.0%, Staph1N (JX080300)—96.0%, and Fi200W (JX080303)—95.1%. The genomic comparison of fRuSau02 with phages K and Twort showed identity rates of 93.5% and 46.5%, respectively. Figure 3 shows a BLASTN comparison of the genomes of phages fRuSau02, K and Twort. The whole-genome level nucleotide phylogeny analysis of fRuSau02 and the 34 phage genomes described in Table S3 showed that fRuSau02 clusters in the same species with A5W, Staph1N, MSA6, Fi200W, and 676Z (Figure 5). The analysis yielded average support of 16% and the OPTSIL clustering resulted altogether to 22, 2, and 1 clusters at species, genus, and family levels, respectively.

Most fRuSau02 nucleotide differences to MSA6 were single base pair substitutions or small indels in intergenic regions. There were 25 coding regions having differences to MSA6, eight of which had silent mutations, leading to proteins with 100% amino acid identity with their MSA6 counterparts (terminal repeat encoded protein TreP, phage terminase Ter.2, tail morphogenetic proteins TmpB and TmpG, DNA helicase DhlA, DNA primase/DNA helicase Pri, ribonucleotide reductase NrdE, and intron encoded endonuclease I-MsaI). Ten proteins had difference(s) to the corresponding proteins in MSA6 but showed 100% amino acid identities to their homologs in other Twort-like viruses: terminal repeat encoded protein TreK was identical to phage G1 ORF159 but showed only 87.4% identity to MSA6 TreK. Terminal repeat encoded protein TreG was identical to phage K Gp007, putative membrane protein MbpP to G1 ORF007, hypothetical protein DmcA to Gp122 of phage JD007, putative membrane protein MbpE to phage G1 ORF120, major tail sheath Tsp to phage K Gp166, hypothetical protein RS_209 to Team1 Gp041, putative receptor binding protein RS_126 to SA5 ORF40, terminal repeat encoded protein TreB to A5W TreB and to G1 ORF231, and putative portal protein Prt to G1 ORF014. Each of these proteins had one to two amino acid difference(s) to their MSA6 homologs. Seven proteins had at least one amino acid (according to present knowledge) unique to fRuSau02, i.e., have not been observed in homologous proteins of any other Twort-like virus: putative membrane protein MbpC, tail morphogenetic protein TmpC, putative receptor binding protein RS_124, putative polymerase-associated exonuclease PolA.2, putative RNA polymerase sigma factor Sig, hypothetical protein RS_200, and putative membrane protein MbpI.

Of the “unique” fRuSau02 proteins, RS_124 is of outmost interest. It is 98.1% identical at the amino acid level to the *orf103* gene product of phage *Φ*SA012, shown to be one out of two receptor binding proteins (RBPs) of this phage [22]. Phage fRuSau02 RS_124 has histidine in position 306, where all the other Twort-like viruses sequenced so far have proline. The residue 306 is part of a carbohydrate binding domain, which is formed by amino acids 213–336. Preliminary structural modelling of this region showed that H306 fits nicely into an anti-parallel *β*-sheet structure, which is completely distorted by H306P change (not shown). This suggests that the structure of the receptor binding protein of fRuSau02 may be different from all the other Twort-like viruses characterized so far.

### 3.5. Genes Interrupted by Self-Splicing Elements

The presence of mobile splicing elements in the genomes of Twort-like viruses is a characteristic feature of staphylococcal myoviruses [17,18,19]. In the case of fRuSau02 phage, four protein-encoding genes were found to be interrupted by different insertion sequences (Figure S1): (1) The gene encoding phage lysin (Lys) is fragmented into two by an intron-encoded HNH homing endonuclease gene (*I-KsaI*). (2) The terminase large subunit gene is divided by the intron, encoding I-MsaI, a protein of unknown function. (3) The gene encoding the putative DNA polymerase-associated exonuclease (PolA) contains two introns encoding proteins I-KsaII and I-KsaIII. (4) The gene encoding phage RecA-like recombinase (Rec) contains the intron-encoded endonuclease gene *I-MsaII*. All of the intervening sequences are predicted to be group I introns encoding putative endonucleases. The functionality of the spliced genes was shown by the fact that the presence of full proteins encoded by three out of four spliced genes was observed in the LC-MS/MS analysis. Namely, lysin, terminase large subunit, and phage recombinase were among the proteins identified in the LC-MS/MS analysis of the purified phage particles (Table 1), with peptides present in both the C- and N-terminal parts of the proteins (see below). While this does not conclusively prove that the polypeptides are continuous, this is the most likely option. The gene splicing pattern of lysin and polymerase encoding genes is identical to the one presented by staphylococcal phages G1, K and ISP [19,47,48]. However, fRuSau02 has additional insertions in the terminase large subunit and recombinase genes that were also present in phage Team1. Unlike in the more distant phages Remus/Romulus and Twort, there were no intein domains identified in the fRuSau02 genome [17,49,50].

### 3.6. Proteomic Analysis of the Phage Structural Proteins

To confirm the identification and expression of the phage structural proteins, a proteomic analysis of the purified phage particles using LC-MS/MS was performed. The comparative analysis of obtained peptide sequences with the sequences of predicted phage proteins allowed for the identification of these structural proteins. To exclude the possibility of obtaining false positive results due to similarity with bacterial proteins that could be carried over from the lysate during the sample preparation, the obtained peptide sequences were compared simultaneously against the phage and bacterial protein sequences. Altogether, 81 phage proteins were identified in the LC-MS/MS analysis, of which 78 fulfilled the inclusion criteria (>2 unique peptides and/or >5% coverage) (Table 1). The analysis of the structural proteome identified the capsid (Mcp) and the tail (Tsp, TmpA, TmpB, TmpC, TmpD, TmpE, TmpF, TmpG, TmpH, BmpA, BmpB, BmpC) proteins, receptor binding proteins (RS_124 and RS_126), the portal protein (Prt), and putative membrane proteins (MbpC, MbpD, MbpG, MbpH, MbpR, MbpS), as well as phage holin (HolA). Additionally, this study showed the presence of ribonucleotide reductases (NrdE and NrdF), DNA-binding protein (HmzG), putative ligase (Lig), recombination nuclease B (RncB), ribonuclease H (Rbn), and DNA helicases (DhlA and DhlB) as well as sigma and anti-sigma factors (Sig, Asf). Altogether, 33 of the identified proteins were annotated as novel phage structural proteins. As already described above, the LC-MS/MS analysis showed also the presence of three proteins: the phage lysin (LysK), terminase (Ter) and recombinase (Rec) encoded by the genes interrupted by the intervening sequences. The prohead protease Pro was also identified in the LC-MS/MS analysis. The presence of the protease among the structural proteins was described previously for *Lactobacillus delbrueckii*-specific phages [51,52] but may be the result of lack of dissociation from the head after the assembly.

### 3.7. fRuSau02 Host Range

A collection of 135 *Staphylococcus* strains, including 51 human and 54 porcine *S. aureus* isolates and 30 coagulase-negative *Staphylococcus* strains of human origin were used to assess the host range (Table 2). Of the 50 clinical *S. aureus* strains collected for this study, 35 were methicillin-sensitive and 15 methicillin-resistant (Table S1). Phage fRuSau02 was able to infect 49 (96%) coagulase-positive and 15 (50%) coagulase-negative strains of human origin, whereas the rate of infection of pig isolates was much lower with only 18 (33%) strains infected. The infectivity of *S. aureus* strains did not depend on their response to methicillin. Some of the bacterial strains instead of clear lysis displayed turbid lysis or only slower growth rate. Counting together the clear and turbid lysis, 33 (61%) of pig isolates and 5 (17%) of coagulase-negative strains were resistant to phage infection. Importantly, all *S. aureus* human isolates (including MRSA strains) were at least moderately sensitive to fRuSau02. Further, patient isolates of coagulase-negative *Staphylococcus* strains, including *S. intermedius*, *S. lugdunensis*, *S. epidermidis*, *S. haemolyticus*, *S. saprophyticus* and *S. pseudointer*, showed different rates of infection depending on the strain, however, at least one strain of each species was susceptible to the phage. Further, the coagulase-negative strains and several pig isolates displayed lower efficiency of infection and turbidity of the plaques, while human *S. aureus* isolates were characterized by big (1–3 mm) clear plaques (data not shown).

### 3.8. fRuSau02 Receptor

**Figure 6:**
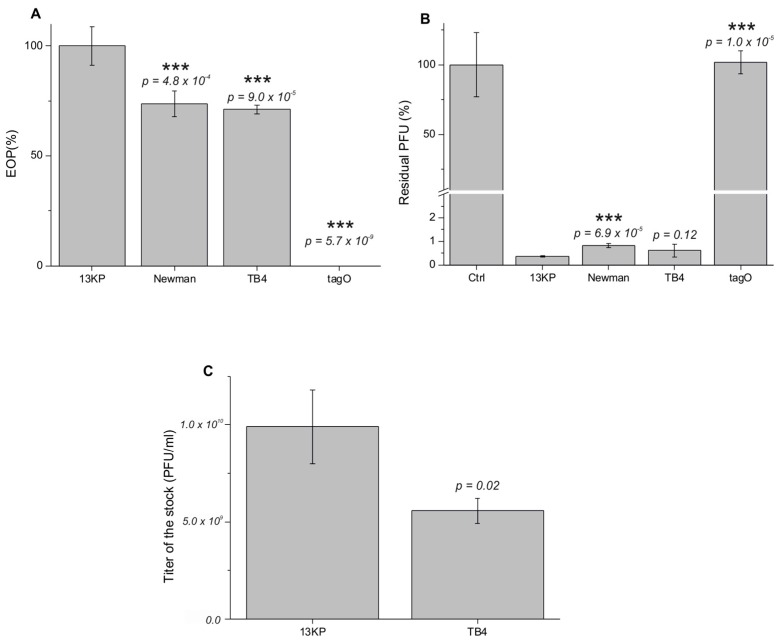
Suitability of host strains for production of fRuSau02. (**A**) The efficiency of plating (EOP) counted as the number of plaque-forming units (PFU) obtained from the same amount of phage lysate for different bacterial strains. The result obtained for the reference strain 13KP was set as 100%; (**B**) Adsorption of fRuSau02 to bacterial surface. Ctrl represents LB as negative control, in which the residual PFU was set to 100%; (**C**) The titer of fRuSau02 lysate produced in strains 13KP and TB4. Error bars indicate SD, *p*-values the level of significance between 13KP and other strains, *** indicates that the difference is statistically significant at the *p*-value < 0.001 level.

Staphylococcal Twort-like phages have been shown to utilize cell wall teichoic acids (WTAs) as their receptors [22]. To test whether this is also the case with fRuSau02, we analyzed the infectivity of fRuSau02 in *S. aureus* strain tagO. This strain carries a mutation in the gene encoding TagO, an enzyme that catalyzes the transfer of *N*-acetylglucosamine to bactoprenol in the first step of teichoic acid biosynthesis [53,54]. As shown in Figure 6A, the phage failed to reproduce in the tagO strain. Furthermore, the adsorption assay revealed that fRuSau02 was not able to adsorb to this strain (Figure 6B). It thus seems that like for other staphylococcal Twort-like phages, WTAs serve as receptors for fRuSau02.

### 3.9. The Choice of Optimal Host Strain for Therapeutic Phage Production

In an ideal situation, host strains used for the production of therapeutic phages should be free of prophages. This is because prophages encode virulence factors, such as staphylococcal enterotoxins [55]. In addition, they can be induced from cells during the infection by therapeutic phage and cause genome variations [56]. To study the possibility to produce fRuSau02 in the prophage-free *S. aureus* strain, we compared the efficiency of plating (EOP) in strains 13KP, Newman, and TB4. Of these, 13KP is the strain that was used as a host strain during the phage isolation, and it was used as a control strain with 100% EOP. TB4 is a prophage-free strain [53] and Newman the parental strain for TB4 [57].

The EOP assay showed that both TB4 and Newman had reduced infectivity compared to the reference strain 13KP (Figure 6A). This was not due to the lowered adsorption efficiency of fRuSau2 to these strains, as the adsorption assay did not reveal significant difference between 13KP and TB4. The phage adsorption to Newman was significantly reduced from 13KP, even though the residual PFU even in this strain was only 0.8% (Figure 6B). The potential of TB4 strain for phage production was further studied by preparing phage stocks. Semi-confluent soft-agar overlay plates were prepared using host-strain adjusted amount of fRuSau02 bacteriophage. Phage stocks were prepared in three parallels and titrated (Figure 6C). The results showed that the difference in the amount of phage obtained using 13KP and TB4 as host was statistically significant (*p* = 0.020), however even the titer obtained in TB4 was sufficient for phage production purposes.

As genes for bacterial toxins often reside in prophage genomes, we wanted to analyze whether fRuSau02 lysate produced in TB4 strain contains less toxins than the lysate produced in 13KP or Newman strains. To this end, the staphylococcal enterotoxins were measured from the phage lysates with the Transia Plate Staphylococcal Enterotoxins assay that detects enterotoxins A, B, C, D, and E. The phage lysate produced in 13KP was clearly positive for enterotoxins, with a concentration that approximately corresponded to 320 ng/mL of staphylococcal enterotoxin A. The phage lysate produced in Newman strain had clearly less toxins (~6 ng/mL) and the lysate produced in TB4 remained negative, indicating that the enterotoxin concentration was lower than the detection limit of the assay (~1 ng/mL of enterotoxin A). It should be noted here that the assay is not validated for quantitative analysis, thus the concentrations need to be considered approximates.

To conclude, strain TB4 should be considered as potential bacterial host for the production of bacteriophage preparations for phage therapy, as it does not possess the risk of temperate phage or enterotoxin carry-over.

## 4. Discussion

This study reports a new bacteriophage, fRuSau02, isolated from a commercial *Staphylococcus* bacteriophage cocktail produced by Microgen. The genomic analysis revealed that fRuSau02 is very closely related to phage MSA6 and many other Twort-like viruses. Bacteriophage fRuSau02 possesses a large genome (148,464 bp) with typical modular organization and a low G+C content and therefore can be classified as a member of the genus *Twortlikevirus*. In coherence, the morphology of the fRuSau02 phage is similar to MSA6 [20], as well as the staphylococcal phage K [19] and *Listeria* phage A511 [58]. The phylogeny analysis of 35 Twort-like phages clustered fRuSau02 and MSA6 in a same species together with A5W, Staph1N, Fi200W, and 676Z. Phage Twort, the type representative of this genus, is more distantly related to fRuSau02, the two phages displaying only 46.5% identity at the nucleotide level. Perhaps the most significant difference between fRuSau02 and MSA6 was the H306P change in the putative receptor binding protein (RS_124 and ORF094 in fRuSau02 and MSA6, respectively). Histidine in position 306 seems unique for fRuSau02, as homologous proteins of other Twort-like viruses analyzed so far, for example G1ORF008 of phage G1, ORF107 of Sb-1, and ORF125 of Team1, all have P306. The preliminary structural modelling indicated the H306P change alters the structure of the carbohydrate-binding region of the RBP, which may have a profound effect on the phage host range.

The in silico analysis revealed the presence of bacterial promoters in the genome of fRuSau02. However, both the presence of genes encoding for the phage sigma and anti-sigma factors and the genomic region of 27.5 kb that does not contain any promoters suggest the existence of phage promoters. Although the performed bioinformatics study failed to reveal any conserved sequences present upstream of the genes of this module, we believe that fRuSau02 possesses two types of promoters. Most likely, in the beginning of the infection process viral genes are transcribed by the bacterial sigma factor. During this step, the sigma and anti-sigma factors encoded by the phage genome are also transcribed. In the later stages of infection, the anti-sigma factor inhibits the activity of the bacterial factor and allows the phage sigma factor to lead the transcription from its own unique promoters. Such a process would allow the bacteriophage to have a high and uniform rate of transcription of late structural genes with a minimal transcription of bacterial genes. Further studies aiming at the recognition of transcriptional starting sites are needed to indicate the possible viral promoters and to validate the annotated bacterial promoters.

The LC-MS/MS analysis revealed 78 phage structural proteins. Due to the high sensitivity of the method, we have to take into consideration the possibility that some of the identified proteins are carried over from the lysate and co-isolated with the phage particles. On the other hand, previous studies showed that some proteins are commonly packed together with the DNA due to their association with nucleic acids [59,60]. For example, the phage sigma and anti-sigma factors (RS_157 and RS_133, respectively) were among the proteins identified, however, it is not likely that they are structural proteins of the phage particles. Earlier studies showed that the primary staphylococcal polymerase *σ*^SA^, directs transcription of early genes in Twort-like viruses [47]. In addition, both bacterial RNA polymeras (RNAP) subunits and the sigma factor were among the bacterial proteins identified in the LC-MS/MS study (data not shown) suggesting that these proteins were co-isolated together with the phage particles. It is possible that these proteins show physical properties that make them either more prone to be co-isolated with phage particles during the purification process or they display unspecific binding to the capsid proteins. Similarly, the proteome analysis showed the presence of putative membrane proteins (MbpC, MbpD, MbpG, MbpH, MbpR, MbpS) that can be a part of the structural proteome used during the infection step or during the assembly and lysis. However, due to the fact that they bind to the membranes, it is also possible that they were carryover from the phage lysate.

Phage fRuSau02 was shown to infect a considerable number of human *S. aureus* isolates, however, the rates of infectivity were much lower among the animal isolates. Similar host range pattern has earlier been observed with phage ISP, which also infects efficiently human *S. aureus* isolates but is unable to infect *S. aureus* strains isolated from pigs [48]. The resistance of the pig strains may be due to minor structural differences of WTA between different *S. aureus* strains. It is also plausible that some strains developed phage resistance without the introduction of modifications in the phage receptor structures. For example, the presence of CRISPR sequences in the genome of *S. aureus* allows the bacteria to acquire the immunity against encountered phages [61]. The pig MRSA strains often belong to only few clonal complexes [62], which may explain their different phage profile compared to *S. aureus* stains isolated from other sources. Interestingly, the host profile of fRuSau02 may be somewhat different from ISP, as fRuSau02 was also able to infect some coagulase-negative staphylococcal strains, including *S. haemolyticus* earlier shown to be resistant for ISP [48].

Phages have important potential as antimicrobial agents and may serve as an alternative to antibiotics, especially in case of multi-drug resistant pathogens. Phage therapy is a possible cure for community-acquired and nosocomial infections caused by drug-resistant *Staphylococcus*, as well as a good candidate for prevention of bacterial contamination in industry and animal husbandry [53,54,55,56,57,58,59,60,61,62,63,64,65,66,67]. Twort-like phages are perhaps the most studied *S. aureus* phage group for clinical applications [15]. For example, phage ISP is one component of a phage therapy cocktail BFC-1, developed for the treatment of burn wound infections [68,69].

To conclude, both our analyses and the fact that fRuSau02 was isolated from a commercial therapeutic phage cocktail suggest that it should be considered as well suited for human phage therapy against coagulase-positive and to some extent also coagulase-negative staphylococcal strains. However, its capacity for the prevention and control of MRSA carriage and/or contamination in the animal husbandry and food industry may be more limited. The efficacy and safety of fRuSau02 as the therapeutic tool is still to be elucidated. Further research that includes pharmacological trials is essential to confirm the possible role of fRuSau02 in the treatment of different forms of MRSA infections in humans.

## Figures and Tables

**Table 1:** The structural proteins of phage fRuSau02 identified using liquid chromatography tandem mass spectrometry (LC-MS/MS).

Locus	Name	No of AA	*M*_W_ * [kDa]	pI * (calc.)
RS_018	TreR, terminal repeat encoded protein R	156	17.8	3.78
RS_033	phage structural protein	105	11.8	6.76
RS_036	phage structural protein	64	7.6	4.65
RS_037	phage structural protein	245	28.6	6.58
RS_041	phage structural protein	57	6.8	5.26
RS_042	phage structural protein	160	18.8	4.64
RS_046	putative membrane protein MbpR	91	10.9	5.01
RS_048	phage structural protein	372	42.2	4.84
RS_050	phage structural protein	138	16.0	5.22
RS_051	HmzG, DNA-binding protein	100	11.3	4.91
RS_055	phage structural protein	87	10.1	5.91
RS_059	Lig, putative DNA or RNA ligase	298	35.0	5.57
RS_061	Phr, putative PhoH-related protein	246	28.6	5.29
RS_063	Rbn, phage ribonuclease H	141	15.8	7.27
RS_067	phage structural protein	75	9.2	9.95
RS_070	putative membrane protein MbpS	263	29.3	8.82
RS_072	LysK.1, phage lysin	209	23.1	9.66
RS_074	LysK.2, phage lysin	267	29.8	9.45
RS_075	HolA, phage holin	167	18.1	4.25
RS_078	DmcB	69	8.0	5.97
RS_080	putative membrane protein MbpC	108	13.0	5.54
RS_082	putative membrane protein MbpD	88	10.3	8.31
RS_085	Ter.1, phage terminase	65	7.7	9.60
RS_087	Ter.2, phage terminase	515	59.7	6.10
RS_088	phage structural protein	266	29.8	5.30
RS_094	Prt, portal protein	563	64.0	6.42
RS_095	Pro, prohead protease	257	28.6	5.01
RS_096	phage structural protein	318	35.9	4.46
RS_097	Mcp, major capsid protein	463	51.2	5.24
RS_098	phage structural protein	98	11.3	9.42
RS_099	phage structural protein	302	34.1	5.24
RS_100	phage structural protein	292	33.7	5.82
RS_101	phage structural protein	206	23.7	10.32
RS_102	phage structural protein	278	31.7	4.79
RS_104	Tsp, major tail sheath protein	587	64.5	4.98
RS_105	TmpA, tail tube protein	142	15.9	5.54
RS_109	phage structural protein	103	12.2	6.13
RS_110	phage structural protein	152	18.1	4.79
RS_111	TmpB, tail morphogenic protein	178	20.9	4.40
RS_112	TmpC, phage DNA transfer protein	1351	143.7	9.11
RS_113	TmpD, tail murein hydrolase	808	91.2	6.74
RS_114	TmpE, putative peptidoglycan hydrolase	295	34.6	4.60
RS_115	Glycerophosphoryl diester phosphodiesterase	848	96.0	4.96
RS_116	phage structural protein	263	29.3	8.19
RS_117	phage structural protein	174	19.9	4.61
RS_118	BmpA, baseplate morphogenetic protein	234	26.6	4.77
RS_119	BmpB, baseplate morphogenetic protein	348	39.2	4.86
RS_120	TmpF, tail morphogenetic protein	1019	116.2	5.08
RS_121	BmpC, baseplate morphogenetic protein	173	19.2	5.39
RS_122	TmpG, tail morphogenetic protein	1152	129.0	5.19
RS_124	receptor binding protein	640	72.6	7.39
RS_126	receptor binding protein	458	50.3	6.27
RS_127	DhlA, DNA helicase	582	67.2	5.85
RS_129	DhlB, DNA helicase	480	54.5	5.72
RS_132	RncB, recombination nuclease B	639	73.4	5.19
RS_133	Asf, anti-sigma factor	198	23.2	6.81
RS_137	phage structural protein	202	23.6	5.72
RS_139	NrdE, ribonucleotide reductase	704	80.1	5.64
RS_140	NrdF, ribonucleotide reductase	349	40.4	4.78
RS_141	phage structural protein	109	12.4	4.68
RS_143	phage structural protein	179	21.1	6.95
RS_152	phage structural protein	423	46.8	4.75
RS_153	Rec.1, phage recombinase	74	7.9	6.61
RS_155	Rec.2, phage recombinase	315	35.7	5.16
RS_157	Sig, sigma factor	220	26.6	5.36
RS_158	phage structural protein	210	23.2	4.84
RS_159	TmpH, phage major tail protein	73	7.9	4.54
RS_160	phage structural protein	86	10.3	5.91
RS_163	putative membrane protein MbpG	122	14.0	5.95
RS_165	phage structural protein	178	20.8	7.47
RS_168	phage structural protein	287	32.3	5.76
RS_169	phage structural protein	243	28.3	5.34
RS_170	phage structural protein	152	17.8	4.98
RS_173	putative membrane protein MbpH	132	15.4	8.94
RS_175	phage structural protein	80	9.4	9.31
RS_181	phage structural protein	98	11.3	7.24
RS_196	phage structural protein	87	9.9	10.05
RS_206	NadV, nicotinamide phosphoribosyltransferase	489	56.1	5.44

* *M*_W_: Molecular weight, pI: Isoelectric point.

**Table 2:** The infectivity of fRuSau02 for different staphylococcal isolates. The details and strain references are listed in supplementary material Table S1.

		fRuSau02 Infectivity							
Bacterial Hosts		Infected *			Intermediate *			Resistant *	
**Coagulase-Positive Human Isolates (*n* = 51)**									
*S. aureus*	49		(96%)	2		(4%)	0		(0%)
**Coagulase-Negative Human Isolates (*n* = 30)**									
*S. intermedius*	0			3			2		
*S. lugdunensis*	1			4			0		
*S. epidermidis*	0			1			4		
*S. haemolyticus*	0			2			3		
*S. saprophyticus*	1			2			2		
*S. pseudointer*	0			4			1		
ALL	2		(7%)	16		(53%)	12		(40%)
**Coagulase-Positive Porcine Isolates (*n* = 54)**									
*S. aureus*	18		(33%)	3		(6%)	33		(61%)

* Infected indicates clear lysis or growth inhibition for spot and liquid assays, respectively, intermediate turbid lysis or slower growth rate, and resistant no infection.

## Supplementary Materials

The following are available online at www.mdpi.com/1999-4915/9/9/258/s1. Table S1: Host range analysis of fRuSau02; Table S2: Putative promoter sequences identified in the fRuSau02 genome; Table S3: Comparative nucleotide analysis between the genomes of fRuSau02 and selected *Staphylococcus* phages; Figure S1: Split genes in the genome of fRuSau02.
