# Supplementary material for: Characterization of vB_SauM-fRuSau02, a Twort-Like Bacteriophage Isolated from a Therapeutic Phage Cocktail
Source: Viruses. 2017 Sep 14;9(9):258. doi: 10.3390/v9090258 (PMC5618024; doi:10.3390/v9090258)
Supplement: Supplementary file 1 [file viruses-09-00258-s001.pdf]

Table S1. Host range analysis of fRuSau02.

Stor. no	Species	Strain code	Origin	Reference	Sensitivity
6305	*S. aureus*	13 S44 S	Human	Merabishvili 2009	+
5511	*S. aureus*	123777	Human (blood)	This work	+
5515	*S. aureus* MRSA	123749	Human	This work	+
5516	*S. aureus* MRSA	123748	Human	This work	+
5523	*S. aureus*	123732	Human (blood)	This work	+
5526	*S. aureus*	123701	Human (blood)	This work	+
5527	*S. aureus*	123700	Human (blood)	This work	+
5528	*S. aureus*	123698	Human (blood)	This work	+
5530	*S. aureus*	123692	Human (blood)	This work	+
5531	*S. aureus*	123690	Human (blood)	This work	+
5535	*S. aureus*	123656	Human (blood)	This work	+
5676	*S. aureus*	13KP	Human	This work	+
5677	*S. aureus*	13US9295	Human (abscessus)	This work	+
5678	*S. aureus*	TS118	Human (skin wound, oper)	This work	+
5679	*S. aureus*	13US9272	Human (skin wound)	This work	+
5680	*S. aureus*	14KP00231	Human (skin infection	This work	+
5858	*S. aureus*	14TS00327	Human (sputum)	This work	+/-
5682	*S. aureus*	14KP00227	Human (skin wound)	This work	+
5683	*S. aureus*	14KP00230	Human (abscessus)	This work	+
5684	*S. aureus*	13TS08032	Human (skin wound, oper)	This work	+
5685	*S. aureus*	13US9307	Human (genital skin)	This work	+
5686	*S. aureus*	13TS8005	Human (finger scar)	This work	+
5687	*S. aureus*	13TS08018	Human (skin wound)	This work	+
5688	*S. aureus*	13KP11085	Human (infected scar)	This work	+
5689	*S. aureus*	13KP11092	Human (skin tissue)	This work	+
5690	*S. aureus*	13TS08003	Human (skin wound)	This work	+
5861	*S. aureus*	14KP00197	Human (joint fluid)	This work	+
5692	*S. aureus*	13TS08000	Human (skin scar)	This work	+
5693	*S. aureus*	13TS07988	Human (skin wound oper)	This work	+
5694	*S. aureus*	13US9317	Human (decubitus)	This work	+
5695	*S. aureus*	13TS07988	Human (skin wound oper)	This work	+
5696	*S. aureus* MRSA	14TK301	Human (skin scar)	This work	+
5860	*S. aureus*	14KP00190	Human (abscessus)	This work	+
5698	*S. aureus* MRSA	13WR35425	Human (nose)	This work	+
5699	*S. aureus* MRSA	13WR36113	Human (throat nose)	This work	+
5700	*S. aureus* MRSA	13WR36074	Human (throat nose)	This work	+
5701	*S. aureus* MRSA	13VV06500	Human (blood)	This work	+
5702	*S. aureus* MRSA	14WR1639	Human (throat)	This work	+
5703	*S. aureus* MRSA	14WR1495	Human (throat nose)	This work	+
5704	*S. aureus* MRSA	14MR449	Human (throat)	This work	+
5705	*S. aureus* MRSA	14KP175	Human (abscessus)	This work	+
5859	*S. aureus*	14TS00320	Human (skin scar)	This work	+
5849	*S. aureus* MRSA	14WR3359	Human (throat)	This work	+
5850	*S. aureus* MRSA	14WR05292	Human (throat)	This work	+
5851	*S. aureus* MRSA	13ET802	Human (skin scar)	This work	+/-
5852	*S. aureus* MRSA	13KK673	Human (conjunctiva)	This work	+
5853	*S. aureus*	14TS85	Human (skin scar)	This work	+
5854	*S. aureus*	14US150	Human (skin wound oper)	This work	+
5855	*S. aureus*	14US162	Human (skin wound)	This work	+
5856	*S. aureus*	14KP00080	Human (skin tissue)	This work	+
5857	*S. aureus*	14KP00159	Human (skin scar)	This work	+
6248	*S. aureus* MRSA	7879_1_5	Pig	[62]	+
6249	*S. aureus*	7879_6_10P	Pig	[62]	+
6250	*S. aureus* MRSA	7801_1_5	Pig	[62]	-

6251	*S. aureus* MRSA	7065_16_20	Pig	[62]	+
6252	*S. aureus* MRSA	7065_6_10P	Pig	[62]	-
6253	*S. aureus* MRSA	7936_6_10	Pig	[62]	-
6254	*S. aureus* MRSA	7936_11_15	Pig	[62]	-
6255	*S. aureus* MRSA	7936_16_20	Pig	[62]	-
6256	*S. aureus* MRSA	4507_1_5	Pig	[62]	-
6257	*S. aureus* MRSA	4507_6_10	Pig	[62]	-
6258	*S. aureus* MRSA	1333_1_5	Pig	[62]	+
6259	*S. aureus* MRSA	1333_6_10	Pig	[62]	-
6260	*S. aureus* MRSA	1333_11_15	Pig	[62]	-
6261	*S. aureus* MRSA	6277_1_5	Pig	[62]	-
6262	*S. aureus* MRSA	7594_1_5	Pig	[62]	-
6263	*S. aureus* MRSA	7594_6_10	Pig	[62]	-
6264	*S. aureus* MRSA	7594_11_15	Pig	[62]	+/-
6265	*S. aureus* MRSA	7594_16_20	Pig	[62]	-
6266	*S. aureus* MRSA	1057_1_5	Pig	[62]	+
6267	*S. aureus* MRSA	1057_6_10	Pig	[62]	+
6268	*S. aureus* MRSA	1057_11_15	Pig	[62]	-
6269	*S. aureus* MRSA	1057_16_20	Pig	[62]	-
6270	*S. aureus* MRSA	7502_1_5	Pig	[62]	+
6271	*S. aureus* MRSA	7502_6_10	Pig	[62]	+/-
6272	*S. aureus* MRSA	7502_11_15	Pig	[62]	+/-
6273	*S. aureus*	7502_1_5P	Pig	[62]	-
6274	*S. aureus* MRSA	6161_1_5	Pig	[62]	-
6275	*S. aureus* MRSA	6161_6_10	Pig	[62]	-
6276	*S. aureus* MRSA	6161_11_15	Pig	[62]	-
6277	*S. aureus* MRSA	6161_16_20	Pig	[62]	-
6278	*S. aureus*	6161_6_10P	Pig	[62]	-
6279	*S. aureus* MRSA	3582_6_10	Pig	[62]	+
6280	*S. aureus* MRSA	3582_11_15	Pig	[62]	-
6281	*S. aureus* MRSA	0812_1_5	Pig	[62]	+
6282	*S. aureus* MRSA	0812_6_10	Pig	[62]	+
6283	*S. aureus* MRSA	0812_11_15	Pig	[62]	+
6284	*S. aureus* MRSA	0812_16_20	Pig	[62]	+
6285	*S. aureus* MRSA	0250_1_5	Pig	[62]	+
6286	*S. aureus* MRSA	0250_6_10	Pig	[62]	+
6287	*S. aureus* MRSA	0250_11_15	Pig	[62]	+
6288	*S. aureus* MRSA	0250_16_20	Pig	[62]	+
6289	*S. aureus* MRSA	5105_1_5	Pig	[62]	-
6290	*S. aureus* MRSA	5105_6_10	Pig	[62]	-
6291	*S. aureus* MRSA	5105_11_15	Pig	[62]	-
6292	*S. aureus* MRSA	5105_16_20	Pig	[62]	-
6293	*S. aureus* MRSA	0186_1_5	Pig	[62]	+
6294	*S. aureus* MRSA	0186_6_10	Pig	[62]	-
6295	*S. aureus* MRSA	0186_11_15	Pig	[62]	-
6296	*S. aureus* MRSA	6672_1_5	Pig	[62]	-
6297	*S. aureus* MRSA	6672_6_10	Pig	[62]	-
6298	*S. aureus* MRSA	6672_11_15	Pig	[62]	-
6299	*S. aureus* MRSA	1724_1_5	Pig	[62]	+
6300	*S. aureus* MRSA	1724_6_10	Pig	[62]	-
6301	*S. aureus* MRSA	1724_11_15	Pig	[62]	-
6209	*S. intermedius*	T-28101	Human (skin scar)	This work	-

6210	*S. intermedius*	T-31387	Human (conjunctiva)	This work	+/-
6211	*S. intermedius*	T-44583	Human (wound)	This work	+/-
6212	*S. intermedius*	T-101658	Human (skin scar)	This work	-
6213	*S. intermedius*	T-102288	Human (skin scar)	This work	+/-
6214	*S. lugdunensis*	T-121906	Human (conjunctiva)	This work	+/-
6215	*S. lugdunensis*	T-121711	Human (skin tissue)	This work	+/-
6216	*S. lugdunensis*	T-121358	Human (blood)	This work	+
6217	*S. lugdunensis*	T-112973	Human (skin tissue)	This work	+/-
6218	*S. lugdunensis*	T-117019	Human (skin wound oper)	This work	+/-
6219	*S. epidermidis*	T-123224	Human (blood)	This work	-
6220	*S. epidermidis*	T-123049	Human (blood)	This work	+/-
6221	*S. epidermidis*	T-121990	Human (blood)	This work	-
6222	*S. epidermidis*	T-124964	Human (blood)	This work	-
6223	*S. epidermidis*	T-124793	Human (blood)	This work	-
6224	*S. haemolyticus*	T-105994	Human (blood)	This work	+/-
6225	*S. haemolyticus*	T-106117	Human (blood)	This work	-
6226	*S. haemolyticus*	T-107035	Human (blood)	This work	-
6227	*S. haemolyticus*	T-107311	Human (blood)	This work	-
6228	*S. haemolyticus*	T-109765	Human (blood)	This work	+/-
6229	*S. saphrophyticus*	T-52269	Human (urine)	This work	+/-
6230	*S. saphrophyticus*	T-52270	Human (urine)	This work	-
6231	*S. saphrophyticus*	T-52271	Human (urine)	This work	+
6232	*S. saphrophyticus*	T-52272	Human (urine)	This work	-
6233	*S. saphrophyticus*	T-52273	Human (urine)	This work	+/-
6234	*S. pseudointer*	P-264	Human	This work	-
6235	*S. pseudointer*	P-266	Human	This work	+/-
6236	*S. pseudointer*	P-272	Human	This work	+/-
6237	*S. pseudointer*	P-320	Human	This work	+/-
6238	*S. pseudointer*	P-351	Human	This work	+/-

Table S2. Putative promoter sequences identified in the fRuSau02 genome.

Location	*p*-value	Motif site
**Putative promoters with the consensus sequence**		
147377:147405	2.84e-10	TTGACATCCTAACATATAGATGGTAATAT
6989:7017	2.84e-10	TTGACATCCTAACATATAGATGGTAATAT
92209:92237	2.14e-9	TTGACATTTTATATGTTAGGTGGTATAAT
144553:144581	3.61e-9	TTGACACCTTACAAGATACATGTTATTAT
4165:4193	3.61e-9	TTGACACCTTACAAGATACATGTTATTAT
23470:23498	9.31e-9	TTGACAATAGTATCATAATATGATATAAT
31800:31828	1.08e-8	TTGACTTCATAAGTTAACTATGCTATAAT
146641:146669	1.89e-8	TTGACAGTCACTTGAAACCATGATATTAT
79278:79306	1.89e-8	TTGACAGAAAGTTAATAATATGGTATACT
6253:6281	1.89e-8	TTGACAGTCACTTGAAACCATGATATTAT
146344:146372	2.80e-8	TTGACAACCTAGAAACAACATGTTAATAT
119199:119227	4.62e-8	TTGACTCTTTTTACTATATATGGTATATT
110108:110136	4.62e-8	TTGACAAATATAAAAAACTATGTTATAAT
109285:109313	4.62e-8	TTGACAATATAGTTAACTTATGTTATACT
146047:146075	6.60e-8	TTGACATTAAGACCGAATTATTATATAAT
5659:5687	6.60e-8	TTGACATTAAGACCGAATTATTATATAAT
116182:116210	8.32e-8	TTGACAATTTATAATATCTATGATACACT
87322:87350	9.33e-8	TTGACTTGAAAAGGATTCTGTGGTATACT
128438:128466	1.17e-7	TTGACACCTTTGTACTTTTGTATTATACT
125192:125220	1.17e-7	TTGACTCTCTTTTTGTTTTATGGTATATT
28587:28615	1.30e-7	TTGACAAATCCCCTTAGTTATGGTATAAT
130039:130067	1.62e-7	TTGACAATTGAGTATACATAGGTTATACT
13156:13184	1.62e-7	TTGACTTTTTTTACTAAGTATGGTAAGAT
147046:147074	1.80e-7	TTGACTTTCAAGCCCTACAATGTTATTAT
6658:6686	1.80e-7	TTGACTTTCAAGCCCTACAATGTTATTAT
67061:67089	2.74e-7	TTGACACAAGAGTAGTATCATAATATACT
123413:123441	3.04e-7	TTGACAGCTCCTATAGTTTATGATATAGT
144043:144071	1.05e-6	TTGACTTTATTATCATATGGTAGTAATAT
143920:143948	1.05e-6	TTGACTTCTGAATAACTATACTGTAATAT
3655:3683	1.05e-6	TTGACTTTATTATCATATGGTAGTAATAT
3532:3560	1.05e-6	TTGACTTCTGAATAACTATACTGTAATAT
148092:148121	1.91e-6	TTACAATCTTTTAGTTTGTATGGTATAAT
33413:33441	1.91e-6	TTGACATAGGTGGTTTTTTATGCTATAGT
7704:7733	1.91e-6	TTACAATCTTTTAGTTTGTATGGTATAAT
21976:22004	2.07e-6	TTGACAAATACAAATACTTGTAATATAAT
36091:36119	3.60e-6	TTGACAACATAATAACTTTCCTATATACT
140813:140842	1.56e-5	TGACAACTATGAAGCGGGTATGCTATAAT
425:454	1.56e-5	TGACAACTATGAAGCGGGTATGCTATAAT
73082:73110	5.42e-5	TATACATACTGAAAAGGAGAGGATAAAAT
14118:14146	7.30e-5	TGTACAAAATATAGTAAAGGAGATAAAAT
**Putative promoters without the consensus sequence**		
35341:35370		TTGAATAATCGGATGGAACAGGAGCAAACT
36160:36187		TTGACTGGGAGGCTAAGGCTATCAAGCT
42782:42809		TTGACAAAATAACAATATATGATATAAT

Table S3. Comparative nucleotide analysis between the genomes of fRu-Sau02 and selected *Staphylococcus* phages. The identity between the genomes was calculated using EMBOSS stretcher [34]

PHAGE	ACC NO	IDENTITY	(%)	GAPS	(%)
**MSA6**	JX080304	148005/148593	99.6 %	479/148593	0.3 %
**A5W**	EU418428	143510/149412	96.0 %	4818/149412	3.2 %
**Staph1N**	JX080300	143528/149514	96.0 %	4917/149514	3.3 %
**Fi200W**	JX080303	144369/151835	95.1 %	6725/151835	4.4 %
**676Z**	JX080302	144046/151962	94.8 %	6896/151962	4.5 %
**Team1**	KC012913	140116/149133	94.0 %	8899/149133	6.0 %
**K**	KF766114	142178/152081	93.5 %	7381/152081	4.9 %
**IME-SA2**	KP687432	139339/149933	92.9 %	10496/149933	7.0 %
**A3R**	JX080301	136230/152029	89.6 %	14576/152029	9.6 %
**812**	KJ206559	135046/153664	87.9 %	6768/153664	10.9 %
**IME-SA118**	KR902361	132173/155972	84.7 %	23730/155972	15 2 %
**Sb-1**	NC_023009	125894/149595	84.2 %	23538/149595	15.7 %
**S25-3**	AB853330	126401/155188	81.5 %	22174/155188	14.3 %
**phiSA012**	AB903967	127225/156816	81.1 %	23074/156816	14.7 %
**MCE-2014**	KJ888149	123098/152991	80.5 %	15611/152991	10.2 %
**phiIPLA-RODI**	KP027446	123980/154211	80.4 %	17610/154211	11.4 %
**GH15**	JQ686190	121655/153346	79.3 %	18422/153346	12.0 %
**S25-4**	AB853331	116725/155993	74.8 %	31399/155993	20.1 %
**IME-SA1**	KP687431	120520/167811	71.8 %	46940/167811	28.0 %
**G1**	NC_007066	103011/184105	56.0 %	81031/184105	44.0 %
**ISP**	FR852584	102948/183819	56.0 %	80835/183819	44.0 %
**phiIPLA-C1C**	KP027447	72476/152750	47.4 %	16075/152750	10.5 %
**phiIBB-SEP1**	KF021268	72137/152458	47.3 %	16524/152458	10.8 %
**JD007**	JX878671	72473/153512	47.2 %	16724/153512	10.9 %
**P108**	KM216423	71917/152968	47.0 %	16665/152968	10.9 %
**Remus**	JX846612	71218/151374	47.0 %	19641/151374	13.0 %
**SA5**	JX875065	71324/152062	46.9 %	18629/152062	12.3 %
**Stau2**	KP881332	78963/168726	46.8 %	55190/168726	32.7 %
**Romulus**	JX846613	70295/150622	46.7 %	21448/150622	14.2 %
**SA11**	JX194239	70756/151831	46.6 %	18872/151831	12.4 %
**Twort**	NC_007021	70003/150623	46.5 %	22076/150623	14.7 %
**IME-SA119**	KR908644	70874/153070	46.3 %	16648/153070	10.9 %
**pSco-10**	KX011028	62101/148924	41.7 %	47398/148924	31.8 %
**SEP9**	KF929199	58011/148703	39.0 %	56525/148703	38.0 %

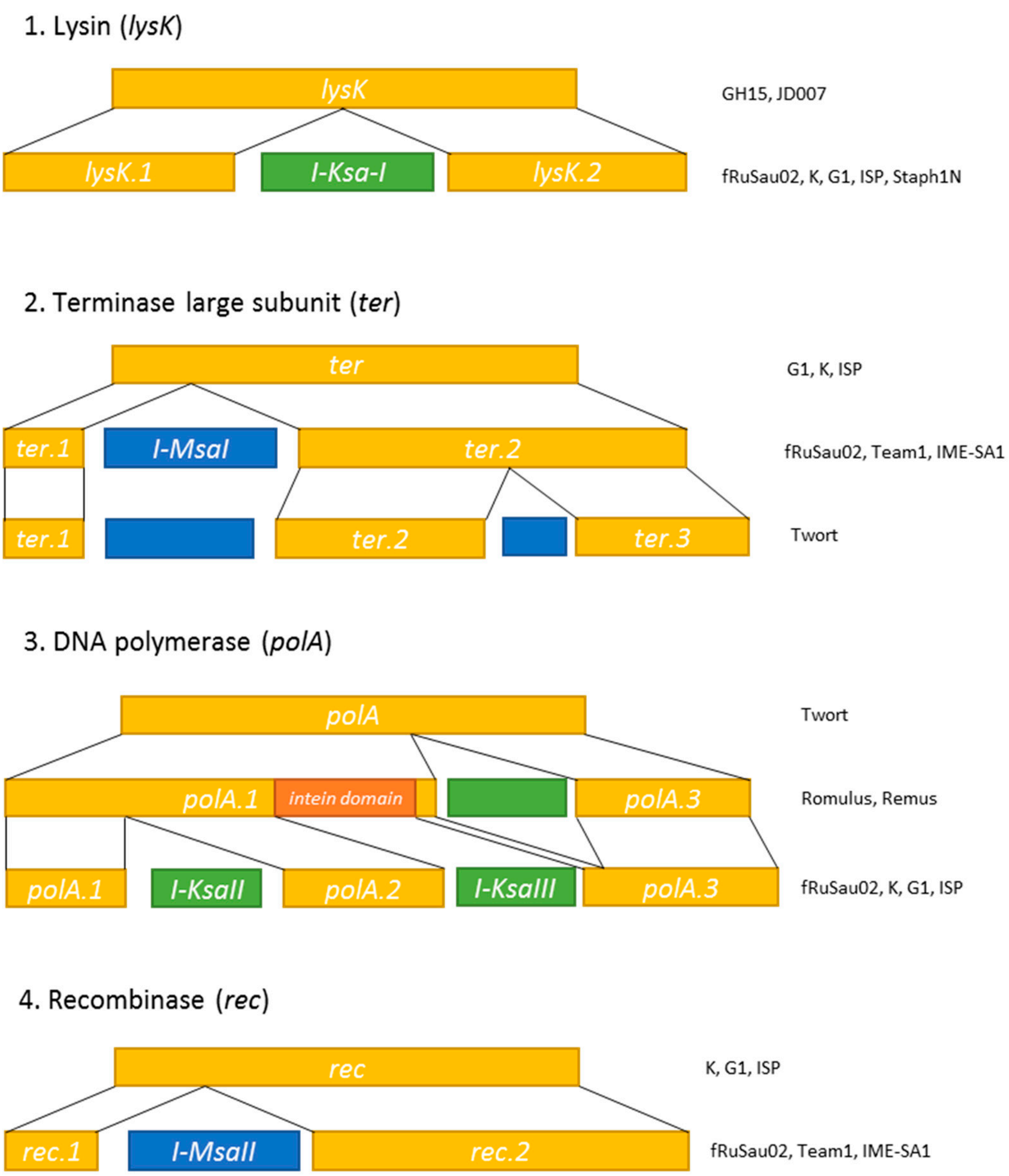

Figure S1. Split genes present in the genome of fRuSau02. The open reading frames are represented as boxes.
